# Chromosome-Scale Genome Assembly of the Hexaploid Taiwanese Goosefoot “Djulis” (*Chenopodium formosanum*)

**DOI:** 10.1093/gbe/evac120

**Published:** 2022-07-26

**Authors:** David E Jarvis, John S Sproul, Beatriz Navarro-Domínguez, Karol Krak, Kate Jaggi, Yung-Fen Huang, Tzu-Yun Huang, Tzu Che Lin, Eric N Jellen, Peter J Maughan

**Affiliations:** Department of Plant and Wildlife Sciences, Brigham Young University, Provo, Utah 84602, USA; Department of Biology, University of Nebraska Omaha, Omaha, Nebraska 68182, USA; Departamento de Genética, Facultad de Ciencias, Universidad de Granada, Granada 18071, Spain; Department of Ecology, Czech University of Life Sciences, Prague 16500, Czech Republic; Department of Plant and Wildlife Sciences, Brigham Young University, Provo, Utah 84602, USA; Department of Agronomy, National Taiwan University, Taipei 10617, Taiwan; Department of Crop Improvement, Taitung District Agricultural Research and Extension Station, Taitung City 950244, Taiwan; Department of Plant Industry, National Pingtung University of Science and Technology, Neipu 91201, Taiwan; Department of Plant and Wildlife Sciences, Brigham Young University, Provo, Utah 84602, USA; Department of Plant and Wildlife Sciences, Brigham Young University, Provo, Utah 84602, USA

**Keywords:** long-read sequencing, chromosome-scale, polyploid evolution, retrotransposon, LTR, *Gypsy*

## Abstract

Djulis (*Chenopodium formosanum* Koidz.) is a crop grown since antiquity in Taiwan. It is a BCD-genome hexaploid (2*n* = 6*x* = 54) domesticated form of lambsquarters (*C. album* L.) and a relative of the allotetraploid (AABB) *C. quinoa*. As with quinoa, djulis seed contains a complete protein profile and many nutritionally important vitamins and minerals. While still sold locally in Taiwanese markets, its traditional culinary uses are being lost as diets of younger generations change. Moreover, indigenous Taiwanese peoples who have long safeguarded djulis are losing their traditional farmlands. We used PacBio sequencing and Hi-C-based scaffolding to produce a chromosome-scale, reference-quality assembly of djulis. The final genome assembly spans 1.63 Gb in 798 scaffolds, with 97.8% of the sequence contained in 27 scaffolds representing the nine haploid chromosomes of each sub-genome of the species. Benchmarking of universal, single-copy orthologs indicated that 98.5% of the conserved orthologous genes for Viridiplantae are complete within the assembled genome, with 92.9% duplicated, as expected for a polyploid. A total of 67.8% of the assembly is repetitive, with the most common repeat being *Gypsy* long terminal repeat retrotransposons, which had significantly expanded in the B sub-genome. Gene annotation using Iso-Seq data from multiple tissues identified 75,056 putative gene models. Comparisons to quinoa showed strong patterns of synteny which allowed for the identification of homoeologous chromosomes, and sub-genome-specific sequences were used to assign homoeologs to each sub-genome. These results represent the first hexaploid genome assembly and the first assemblies of the C and D genomes of the Chenopodioideae subfamily.

**Significance**

The high-quality genome assembly of djulis is the first sequenced hexaploid in the genus *Chenopodium*. The genome is a valuable resource for studying genome evolution in polyploids and will facilitate efforts to characterize, conserve, and improve this nutritious, understudied crop.

## Introduction

The *Chenopodium album* L. aggregate represents a taxonomically complex group whose evolutionary history has largely been shaped by hybridization and polyploidization (Mandák et al. 2018; Walsh et al. 2015; Štorchová et al. 2015). The variation in ploidy level includes taxa with diploid (2*n* = 18), allotetraploid (2*n* = 36), allohexaploid (2*n* = 54), and decaploid (2*n* = 90) chromosome counts (Mandák et al. 2016). Plants of this group have been domesticated as seed and/or vegetable crops at various times and places, mostly from weeds that are notorious colonizers of human-disturbed environments, like *C. album* L.. Fortuitously, these plants tend to be highly nutritious, with favorable levels of mineral nutrients and vitamins along with excellent amino acid profiles for their seed and foliar proteins (Yadav and Sehgal 2002; Repo-Carrasco et al. 2003; Vega-Gálvez et al. 2010; Pandey and Gupta 2014). Even as free-living weeds, *Chenopodium* species have been gathered as potherbs throughout human history (Huai and Pei 2000). The increasing popularity of the South American-native pseudocereal quinoa (*C. quinoa* Willd., 2*n* = 36, AABB sub-genomes) has aroused interest in its cultivated cousins, among them Andean cañahua or kañiwa [*C. pallidicaule* Aellen, 2*n* = 18, AA (Mangelson et al. 2019)], Mesoamerican huauzontle [*C. berlandieri* ssp. *nuttaliae* (Safford) H.D. Wilson and Heiser, 2*n* = 36, AABB; (Wilson and Jr 1979)], Himalayan bathua [*C. giganteum* D. Don, 2*n* = 54, BBCCDD; (Partap and Kapoor 1985)], and Taiwanese djulis or hangli [*C. formosanum* Koidz., 2*n* = 54, BBCCDD; (Chu et al. 2016)].

Taiwanese djulis and Himalayan bathua are hypothesized to have arisen as independent Asian domestications from the cosmopolitan allohexaploid BBCCDD weed, lambsquarters (*C. album* L. [Krak et al. 2016; Mandák et al. 2018; Kolano et al. 2019]). Djulis was traditionally used as a fermentation starter for small grain winemaking by Taiwanese aboriginals (Tsai et al. 2010). Djulis is of particular interest not only as a high-quality food source, but also as a biological pigment source and for its documented medicinal properties, high levels and diversity of secondary metabolites—among them phytosterols and triterpenes (Tsai et al. 2010, 2011; Huang et al. 2019)—and for its potential as a bioenergy source (Yang et al. 2014). Djulis extracts and secondary metabolites demonstrated beneficial effects on cardiovascular diseases like hypertension (Chen et al. 2019); in improving symptoms for Type 2 diabetes patients (Li et al. 2021); in inhibiting formation of colon cancer (Lee et al. 2019); and for curtailing adipogenesis (Chyau et al. 2018). Djulis extract has also traditionally been used as an insecticide (Chio et al. 2013; Chuang et al. 2018). To facilitate characterization and preservation of djulis genetic diversity and to begin to characterize the genetic mechanisms underlying its desirable agronomic and medicinal traits, we present a reference-quality, whole-genome assembly of *C. formosanum* and compare its constituent B, C, and D sub-genomes for genic and repetitive sequence compositions.

## Results and Discussion

### Genome Assembly and Annotation

Cytogenetic analysis confirmed that *C. formosanum* is a hexaploid with 54 chromosomes (supplementary fig. S1, Supplementary Material online). DNA sequencing of the *C. formosanum* genome using three PacBio HiFi cells produced 67.906 Gb in 3.982 million reads with minimum quality scores of Q20 and an average read length of 16.923 kb. Using this data, we estimated a genome size of 1.693 Gb (supplementary fig. S2, Supplementary Material online), indicating that the sequencing data represented approximately 41X coverage. Sequencing reads were assembled into 1,914 contigs with a total length of 1.668 Gb (98.5% of predicted genome size) and a contig N50 of 35.895 Mb (L50 of 17) (supplementary table S1, Supplementary Material online). Contigs were scaffolded with Hi-C into a preliminary scaffold assembly containing 1,872 scaffolds spanning 1.668 Gb (supplementary table S1, Supplementary Material online). Scaffold PGA_823_1_5040, the shortest sequence in the assembly, could not be classified by either Kraken or BlobTools (supplementary table S2, Supplementary Material online) and was therefore removed. We also assembled the complete *C. formosanum* chloroplast sequence, spanning 152,194 bp (supplementary fig. S3, Supplementary Material online). We removed 1,052 scaffolds that shared ≥99% sequence identity with the assembled chloroplast over ≥99% of the total scaffold length. We also removed 21 scaffolds totaling 652,248 bp that were identified as mitochondrial sequence by NCBI, resulting in a final, cleaned genome assembly of 798 scaffolds spanning 1.630 Gb (supplementary table S1, Supplementary Material online).

**Fig. 1.**
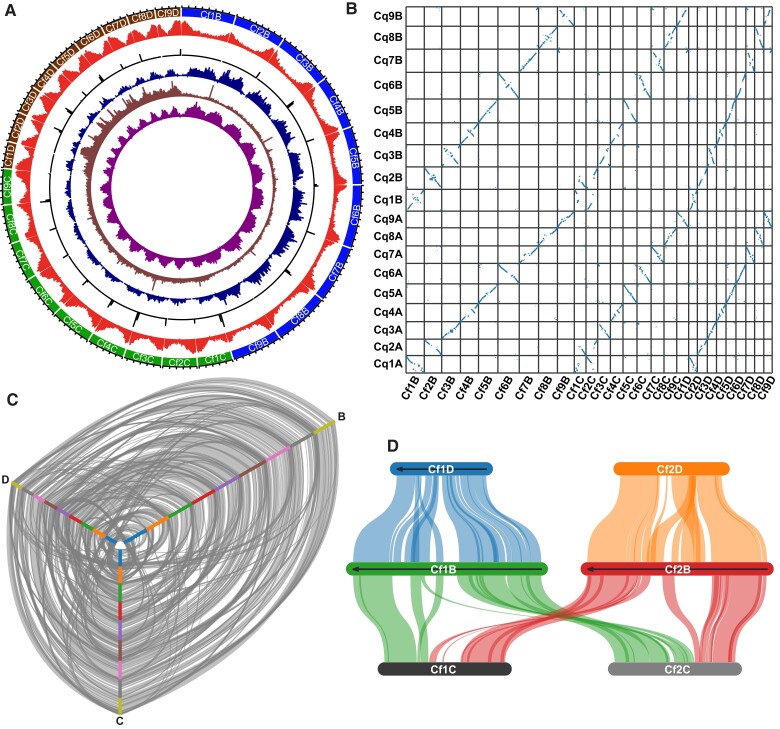
(*A*) Circular representation of the nine chromosomes in each of the *B* (outer track, blue), *C* (green), and *D* (brown) sub-genomes of *C. formosanum*. Tick marks represent 10 Mb. From inside to outside, tracks represent the density of *Gypsy* LTR elements, mapped *C. acuminatum* reads, 18–24J repeats, telomeric repeats, and genes. (*B*) Dotplot visualization of syntenic genes between the genomes of quinoa (*y*-axis) and *C. formosanum* (*x*-axis). (*C*) Hive plot visualization of syntenic genes among the *B, C*, and *D* sub-genomes of *C. formosanum*. For the inside to the outside, chromosomes of each sub-genome are arranged in order from 1–9. (*D*) Detailed view of the syntenic relationships between chromosomes 1 and 2 of the *B, C*, and *D* sub-genomes of *C. formosanum*.

The 27 largest chromosome-scale scaffolds, representing the haploid chromosome number, contain 97.8% of the total assembly length (supplementary fig. S4, Supplementary Material online). Nine of these scaffolds consist of a single contig (supplementary fig. S4, Supplementary Material online). The chromosomes appear to be largely complete, with 24 chromosomes showing an abundance of telomeric repeats (10 at both ends and 14 at one end, fig. 1*A*). A Benchmarking of universal, single-copy orthologs (BUSCO) analysis likewise indicated that the genome assembly is largely complete, with 97.7% and 98.5% of BUSCO genes identified as complete in the Embryophyta and Viridiplantae datasets, respectively (supplementary fig. S5, Supplementary Material online). Not surprisingly for a polyploid genome, 91.1% and 92.9% of the genes in the Embryophyta and Viridiplantae datasets, respectively, were identified as duplicates in the *C. formosanum* genome.

A total of 67.8% of the *C. formosanum* genome assembly was annotated as repetitive by RepeatMasker (supplementary table S3, Supplementary Material online), similar to the repetitive fraction predicted by GenomeScope (65.6%, supplementary fig. S2, Supplementary Material online). The most abundant categorized repeats are *Gypsy* (22.28%) and *Copia* (7.04%) long terminal repeats (LTRs), with 28.90% unknown. To facilitate gene annotation, 3.021 million PacBio Iso-Seq reads with a minimum quality score of Q20 were produced from stems, leaves, petioles, and inflorescences, and reads were assembled into 207,192 high-quality, full-length, non-concatemer transcripts (supplementary table S4, Supplementary Material online). Gene annotation using the assembled transcripts as evidence identified 75,056 protein-coding genes. Gene density is low in centromeric regions and greater toward the chromosome ends (fig. 1*A*).

Comparison of gene synteny with *C. quinoa* enabled the identification of homoeologous sets of *C. formosanum* chromosomes, with three chromosomes showing clear evidence of synteny with each *C. quinoa* chromosome (fig. 1*B*); To assign chromosomes within each homoeologous set to the B, C, or D sub-genome, we first identified and plotted the position of the 18–24J microsatellite repeat, which was previously shown to be overrepresented in the *Chenopodium* B sub-genomes (Kolano et al. 2011). Nine *C. formosanum* chromosomes showed a clear overrepresentation of the 18–24J repeat (fig. 1*A*) and were assigned to the B sub-genome. To identify chromosomes belonging to the D sub-genome, we mapped and visualized the location of sequencing reads from the D-genome diploid *C. acuminatum*. Nine chromosomes showed a clear overabundance of mapped *C. acuminatum* reads (fig. 1*A*) and were assigned to the D sub-genome. The remaining nine chromosomes were assigned to C sub-genome. Using this approach, each homoeologous set of three chromosomes was unambiguously assigned a B, C, and D chromosome. The three sub-genomes displayed a high degree of synteny (fig. 1*C*), although there is clear evidence of large structural rearrangements, including a translocation between Cf1C and Cf2C (fig. 1*D*).

### Repeat Composition

**Fig. 2.**
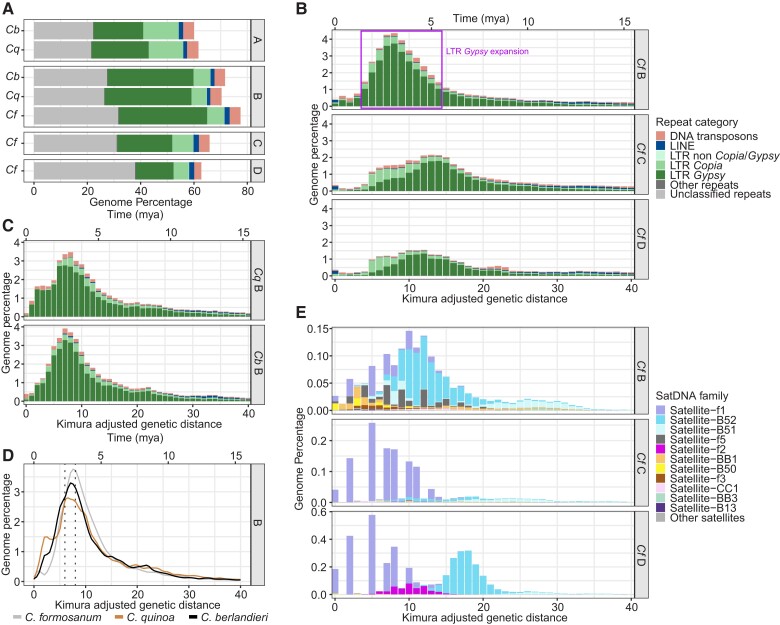
(*A*) Abundance of repeats in the major repetitive element categories for the *A, B, C*, and *D* sub-genomes in *C. quinoa* (*Cq*), *C. berlandieri* (*Cb*), and *C. formosanum* (*Cf*). (*B*) Repetitive element landscape plots for *C. formosanum* sub-genomes. The *y*-axis shows TE abundance as a percentage of the genome for major repeat categories. The *x*-axis shows both sequence divergence (CpG adjusted Kimura distance) relative to consensus sequences for TE superfamilies (below) and an estimate of evolutionary time calculated using LTR sequence evolution rate estimates in rice. (*C*) Repeat landscapes for the B sub-genome in quinoa and *C. berlandieri*. (*D*) Repeat landscape isolating LTR *Gypsy* elements in *C. formosanum*, *C. quinoa*, and *C. berlandieri*. (*E*) Repeat landscape of satellite DNAs in sub-genomes of *C. formosanum*.

We noted substantial differences in sub-genome size, with all B-genome chromosomes larger than all C chromosomes, which are in turn all larger than all D chromosomes (fig. 1*A*, supplementary table S5, Supplementary Material online). B sub-genome chromosomes comprise 45.95% of the total chromosome length, whereas C and D subgenomes comprise 31.22% and 22.82%, respectively. Despite this more than two-fold difference in sub-genome size, the number of non-repetitive bases was similar across sub-genomes (210 Mb, 197 Mb, and 174 Mb in B, C, and D, respectively), suggesting repetitive elements account for most of the size variation across sub-genomes. Repetitive elements comprised 71.3%, 60.3%, and 52.2% of the B, C, and D sub-genomes, respectively (fig. 2*A*). LTRs *Gypsy* elements were the most abundant classified repeats in all three sub-genomes (fig. 2*A*) and were particularly abundant in B sub-genome chromosomes (fig. 1*A*). Repeat landscape plots revealed an apparent historical expansion of *Gypsy* elements in the B subgenome centered at *K* = 7 sequence divergence (fig. 2*B*). The abundance of *Gypsy* elements in the B subgenome amount to ∼137 Mb of additional LTR sequences compared to the next largest sub-genome (C), and accounts for ∼58% of the difference in total assembly length between the two.

An equivalent analysis in the B sub-genomes of *C. quinoa* and *C. berlandieri* also showed major *Gypsy* expansions centered at *K* = 7 (figs. 2*B–D*), suggesting the expansion occurred in the ancestral B sub-genome prior to the origin of the allopolyploids. The *Gypsy* element peak in the B sub-genomes from all three species dates from 2.3–3 million years ago (mya) (figs. 2*B–D*). This timing roughly corresponds with the estimated origin of the B genome (Mandák et al. 2018). We also identified 18 satellite DNA (satDNA) families in the *C. formosanum* assembly, 11 of which are novel, with the three sub-genomes differing in both overall satDNA abundance and family composition, in accordance with previous analyses of the B and D sub-genomes (Belyayev et al. 2020) (fig. 2*E*).

## Materials and Methods

### Genome Assembly and Annotation

DNA extraction, PacBio sequencing, whole-genome and transcriptome assembly, and gene and repeat annotation were performed as previously described (Jarvis et al. 2022), with the exception that EST and protein evidence came from *C. quinoa* (NCBI BioProject PRJNA394242) and *Beta vulgaris* (BioProject PRJNA413079). All protein-coding genes shorter than 50 amino acids and without a hit to the uniport-sprot database were removed. Hi-C scaffolding was performed by Phase Genomics. Genome size was estimated using GenomeScope (Ranallo-Benavidez et al. 2020). Genome, transcriptome, and proteome completeness was assessed using BUSCO (Simão et al. 2015). Putative contaminant sequences were identified using Kraken 2 (Wood et al. 2019) and BlobTools (Laetsch and Blaxter 2017).

To assemble the chloroplast, PacBio reads were mapped to the *C. quinoa* chloroplast genome (GenBank acc: MK159176) using minimap2 (v.2.24) (Li 2018) with the “map-hifi” argument. Samtools v1.9 (Danecek et al. 2021) was used to identify mapped reads with a minimum query length (mlen) >8000, query value (qval) >40, and GC content between 32–40%. These reads were included in a final assembly of the chloroplast genome using HiCanu v2.1 (Nurk et al. 2020) using default parameters, and the assembly was annotated using GeSeq (Tillich et al. 2017). Scaffolds from the genome assembly with sequence homology to the assembled chloroplast were identified by performing a nucleotide BLAST search of the genome assembly against the chloroplast sequencing using default parameters. All scaffolds that shared ≥99% sequence identity with the assembled chloroplast over ≥99% of the total scaffold length were removed from the genome assembly.

### Genome Visualization and Comparison

The positions of *Gypsy* LTRs were determined using RepeatModeler v2.0.1 (Flynn et al. 2020) and RepeatMasker v4.1.0 (Smit et al. 2013), as described below. The positions of 18–24J repeats and telomeric repeats were determined using BLAST, as previously described (Jarvis et al. 2017). The positions of mapped sequencing reads from *C. acuminatum* were determined by generating PacBio Hifi reads from one cell using DNA extracted from roots of a single *C. acuminatum* plant and then mapping the reads to the *C. formosanum* genome assembly using minimap2 (Li 2018) with the “-ax map-pb” option. The densities of *Gypsy* LTRs, mapped *C. acuminatum* reads, 18–24J repeats, telomeric repeats, and genes were visualized in 500 kb windows using Circa (http://omgenomics.com/circa).

Syntenic genes within the *C. formosanum* genome and between the *C. formosanum* and *C. quinoa* genomes were identified using the CoGe SynMap (Lyons et al. 2008) tool and MCScanX (Wang et al. 2012) and visualized using SynVisio (Bandi and Gutwin).

### Repetitive Element Identification and Annotation

Repetitive elements were identified *de novo* in the *C. formosanum* assembly using RepeatModeler v2.0.1 (Flynn et al. 2020) and annotated in each *C. formosanum* sub-genome separately with RepeatMasker v4.1.0 (Smit et al. 2013). Prior to sub-genome annotation we merged the custom repeat library from RepeatModeler with RepeatMasker’s internal “Viridiplantae” library and used the resulting combined library for annotation of sub-genomes with the search engine set to “ncbi” and using the -xsmall option. For comparative analyses that included *C. berlandieri* (genomevolution.org, CoGe id62441) and *C. quinoa* (CoGe id60716) assemblies, the same repeat annotation methods were used. We summarized repeat abundance in each sub-genome by parsing output from repeat annotation software using custom scripts and generating plots in R v3.5.1 (Team 2018).

### satDNA Identification and Annotation

To identify novel satDNA families, we used RepeatMasker to mask previously described (Belyayev et al. 2020) satDNAs in the *C. formosanum* assembly. The repeat masked *C. formosanum* sequence was then used as input for TRF v 4.09.1 (Benson 1999). TRF output files were parsed using custom shell scripts. We selected candidate satDNA sequences based on their abundance (“Copy_number” >50) and monomer length (“Consensus_size” >12 bp). Monomer consensus sequences of the selected satDNA candidates were checked and apparent microsatellite sequences were excluded from further analyses. The remaining candidates were sorted according to the size of monomer consensus sequences. Monomers with similar sizes were aligned together using MAFFT (Katoh et al. 2002) as implemented in Geneious 11.1.5 (Biomatters Ltd, Auckland, New Zeland), to search for sequence homology among them. In case of significant homology, the monomers consensi were grouped together and a new consensus sequence was created. We then aligned the newly described satDNA families to each other and to those previously described (Belyayev et al. 2020). Both the newly described as well as the previously identified satDNA monomer consensi were mapped onto the *C. formosanum* assembly using Geneious’ “Live Annotate and Predict” function. To account for sequence variation between the different arrays of the same satDNA family in the genome we performed three mappings with similarity thresholds of 90%, 80% and 75%.

### Repetitive Element Landscapes

To visualize the different composition of transposable elements and satDNAs among the three C. formosanum sub-genomes, we generated repeat landscape plots for *Chenopodium* species sub-genomes. We used the calcDivergenceFromAlign.pl built-in tool of RepeatMasker to obtain a histogram of the Kimura 2-Parameter divergence for each element. Next, we transformed the abundance values to express them as genome proportions by dividing the number of aligned nucleotides by the total number of nucleotides in the genome assembly. The resulting histograms (referred to as repeat landscapes) were plotted in R, using ggplot2. To get a rough time scale of transposable element evolution, we transformed to time the divergence values of the x-axis in the repeat landscapes using the equation: time = divergence/2r, where r = 1.3 × 10-8 mutations per site per year, which is the previously estimated rate of LTR element sequence evolution in rice (Ma and Bennetzen 2004).

## Supplementary Material

evac120_Supplementary_Data

## Data Availability

The genome assembly of *C. formosanum* and sequence data for *C. formosanum* and *C. acuminatum* have been deposited at DDBJ/ENA/GenBank under the accession JAMXMD000000000, BioProject ID PRJNA840947 (http://www.ncbi.nlm.nih.gov/bioproject/840947).; the version described in this paper is version JAMXMD010000000. The assembly and annotation for *C. formosanum* are also available at CoGe (https://genomevolution.org/coge/) id64369.
