# Supplementary material for: Chromosome-Scale Genome Assembly of the Hexaploid Taiwanese Goosefoot “Djulis” (Chenopodium formosanum)
Source: Genome Biol Evol. 2022 Jul 26;14(8):evac120. doi: 10.1093/gbe/evac120 (PMC9356728; doi:10.1093/gbe/evac120)
Supplement: evac120_Supplementary_Data [file evac120_supplementary_data.zip › Supplementary Figures.pdf]

Supplementary Figures

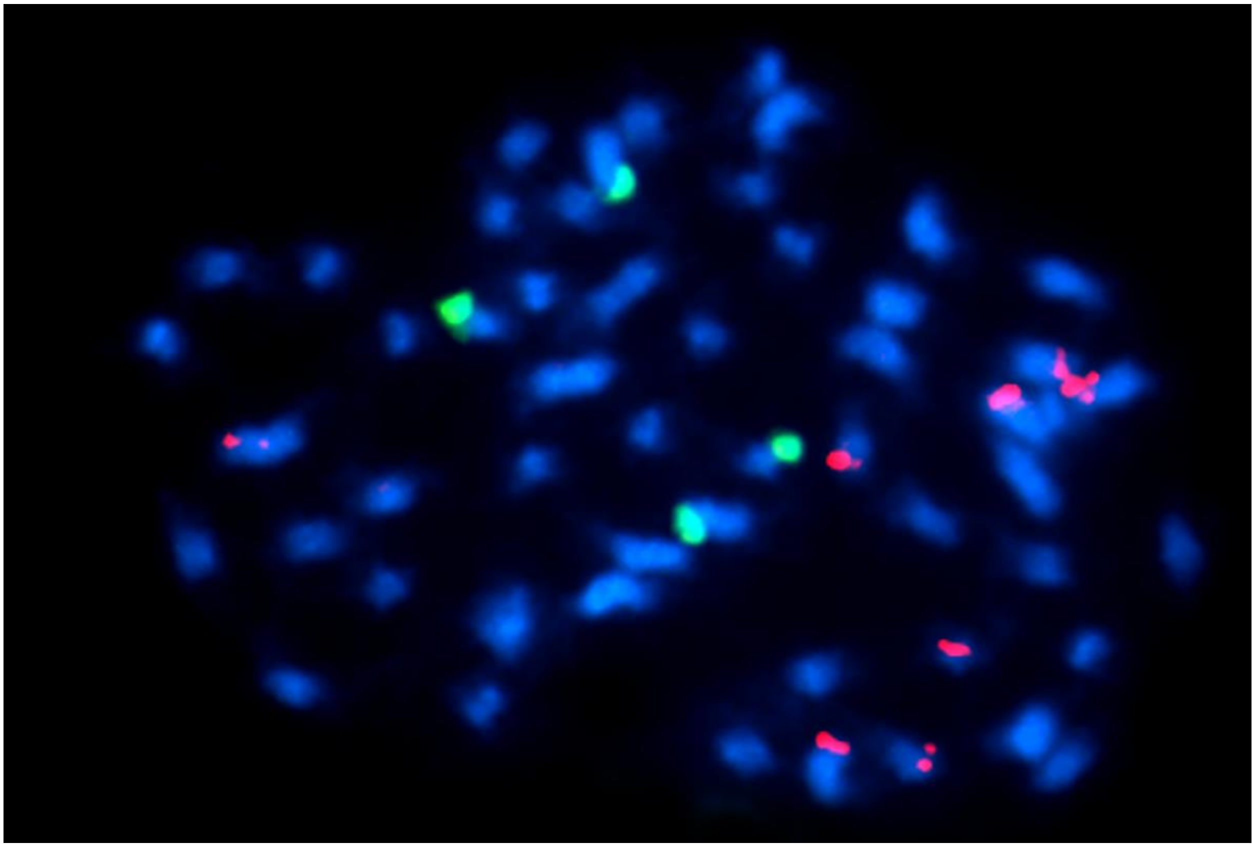

Fig. S1. Somatic metaphase cell of *C. formosanum* hybridized with 35S rRNA (FITC, two loci) and 5S rRNA (Texas Red, four loci) probes and counterstained with DAPI (blue). Magnification is 1000X.

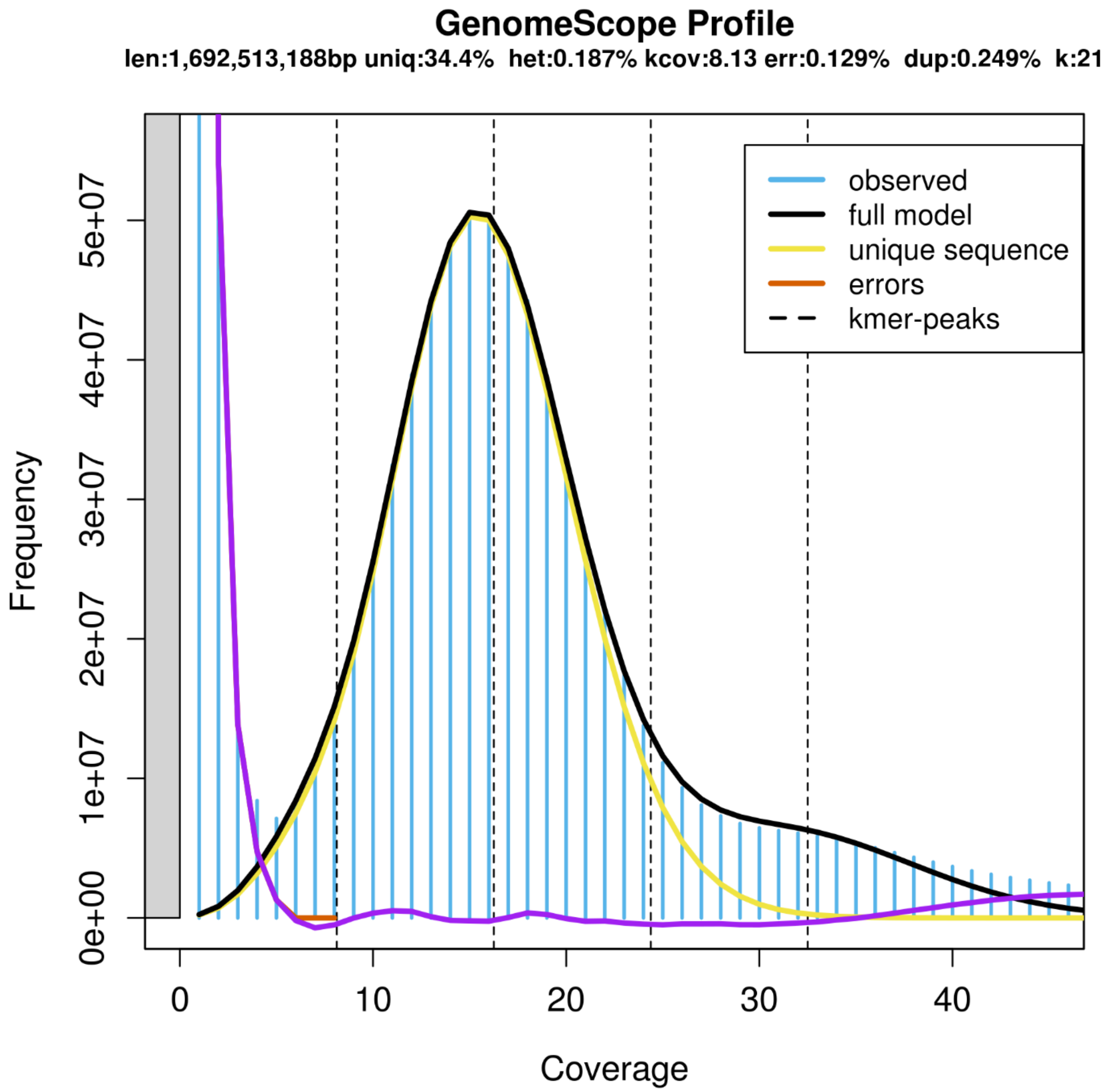

Fig. S2. Estimation of the total genome size and the unique sequence fraction of the *C. formosanum* genome using GenomeScope.

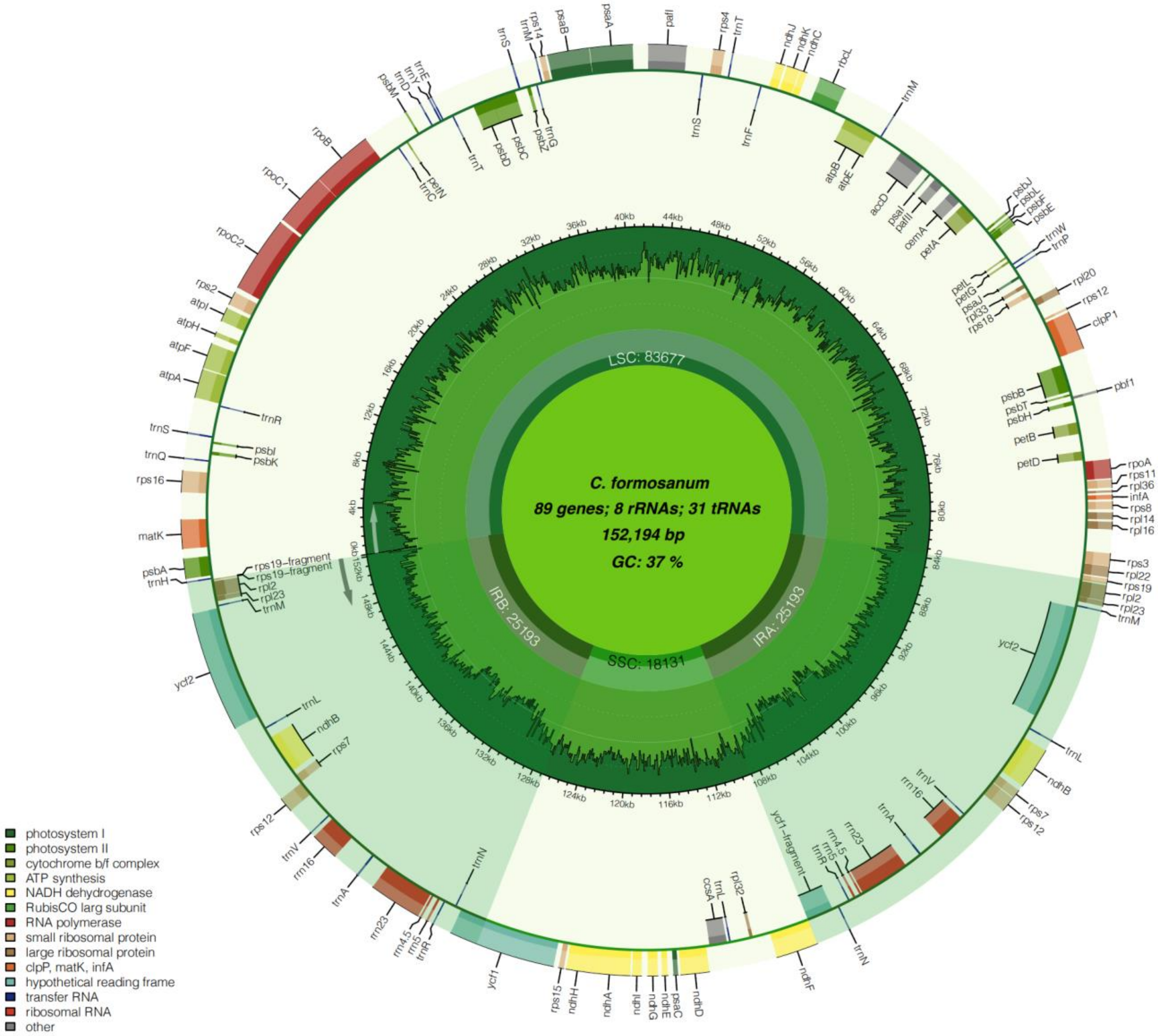

Fig. S3. The assembled and annotated *C. formosanum* chloroplast genome.

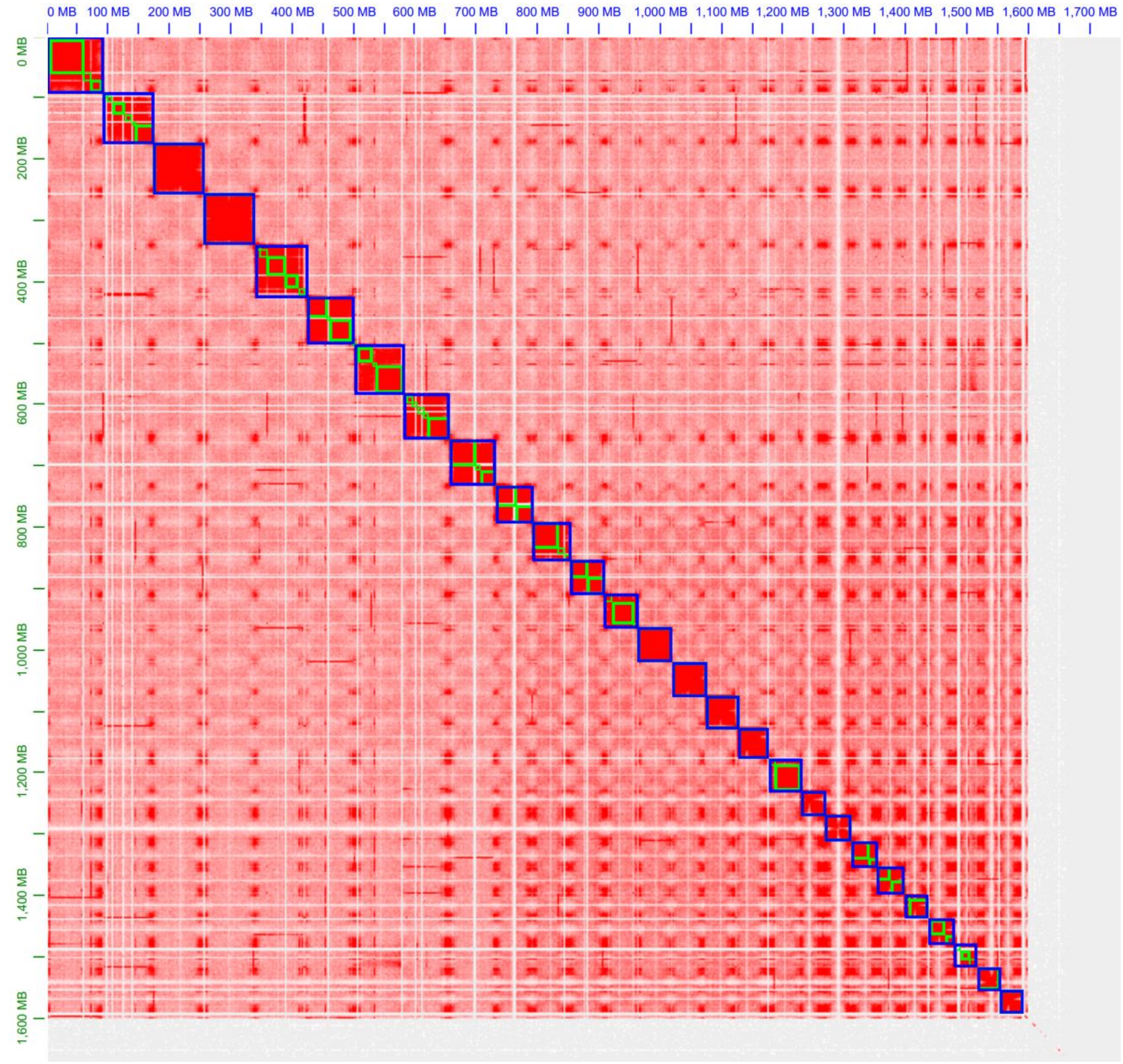

Fig. S4. Hi-C heatmap of sequence contact frequency among the *C. formosanum* contigs (green boxes) assembled into chromosome-scale scaffolds (blue).

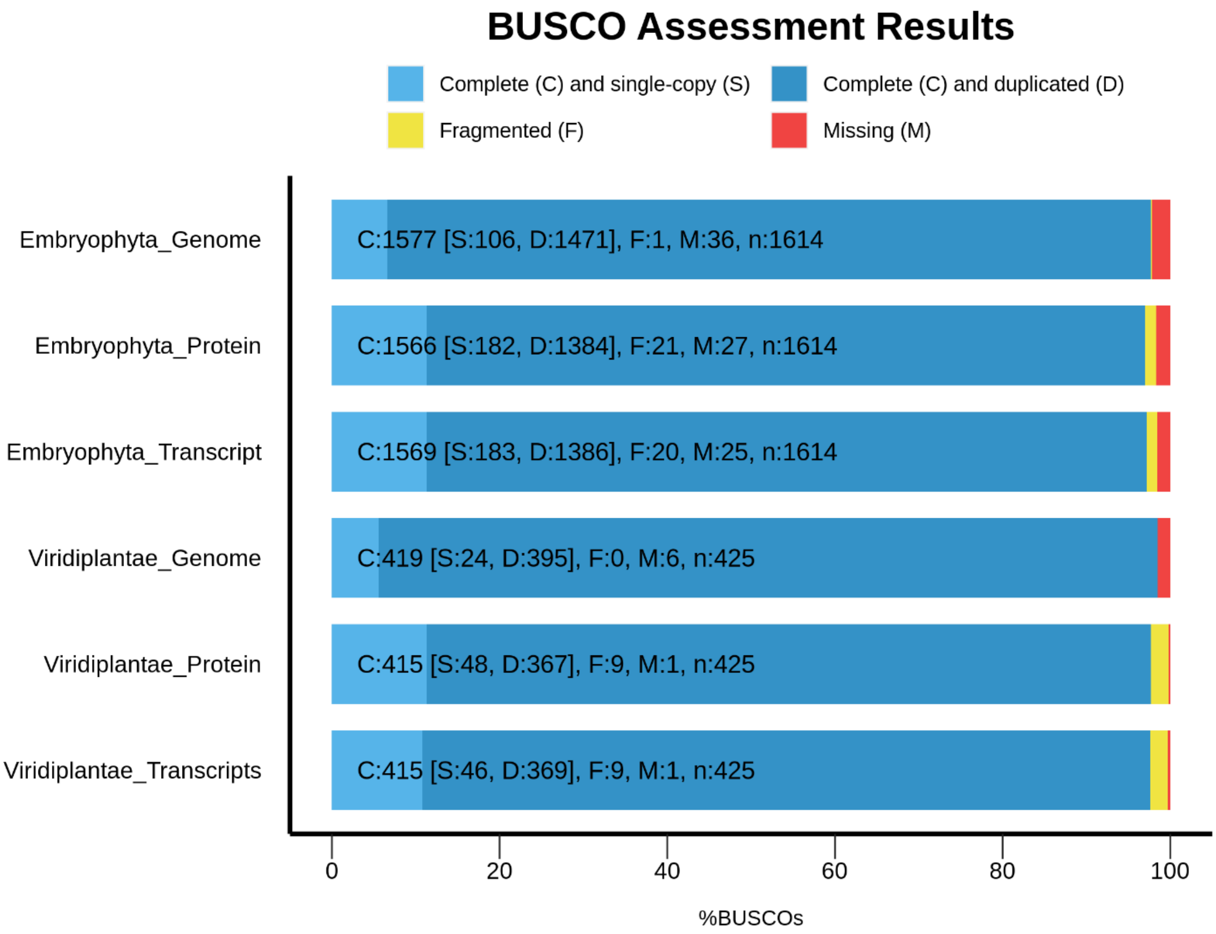

Fig. S5. Assessment of the completeness of the *C. formosanum* genome assembly, predicted protein sequences, and predicted transcript sequences using the Embryophyta or Viridiplantae BUSCO datasets.
